# Identifying likely transmissions in *Mycobacterium bovis* infected populations of cattle and badgers using the Kolmogorov Forward Equations

**DOI:** 10.1038/s41598-020-78900-3

**Published:** 2020-12-15

**Authors:** Gianluigi Rossi, Joseph Crispell, Daniel Balaz, Samantha J. Lycett, Clare H. Benton, Richard J. Delahay, Rowland R. Kao

**Affiliations:** 1 The Roslin Institute and Royal (Dick) School of Veterinary Studies, University of Edinburgh, Easter Bush Campus, Midlothian, EH25 9RG UK; 2 School of Veterinary Medicine, College of Health and Agricultural Sciences, University College Dublin, Beliflied, Dublin, Republic of Ireland; 3 National Wildlife Management Centre, Animal and Plant Health Agency, Woodchester Park, Gloucestershire, UK

**Keywords:** Ecological epidemiology, Phylogeny, Computational models

## Abstract

Established methods for whole-genome-sequencing (WGS) technology allow for the detection of single-nucleotide polymorphisms (SNPs) in the pathogen genomes sourced from host samples. The information obtained can be used to track the pathogen’s evolution in time and potentially identify ‘who-infected-whom’ with unprecedented accuracy. Successful methods include ‘phylodynamic approaches’ that integrate evolutionary and epidemiological data. However, they are typically computationally intensive, require extensive data, and are best applied when there is a strong molecular clock signal and substantial pathogen diversity. To determine how much transmission information can be inferred when pathogen genetic diversity is low and metadata limited, we propose an analytical approach that combines pathogen WGS data and sampling times from infected hosts. It accounts for ‘between-scale’ processes, in particular within-host pathogen evolution and between-host transmission. We applied this to a well-characterised population with an endemic *Mycobacterium bovis* (the causative agent of bovine/zoonotic tuberculosis, bTB) infection. Our results show that, even with such limited data and low diversity, the computation of the transmission probability between host pairs can help discriminate between likely and unlikely infection pathways and therefore help to identify potential transmission networks. However, the method can be sensitive to assumptions about within-host evolution.

## Introduction

In recent years, network models have been increasingly used to represent the complex set of interactions (i.e. contacts) that can lead to pathogen transmission in humans^1,2^, wildlife^3^, and livestock^4,5^. In a network paradigm, individuals or groups of hosts (i.e. farms, social groups, or sub-populations) are represented as *nodes* (in graph theory, *vertices*), and the potential infectious contacts between them as *links (edges)*.

An important distinction exists between *contact network* and *transmission network*: while the former includes all potential transmission contacts, the latter is a subset of the former describing pathogen transmission patterns^5,6^. Identifying the transmission network, even when the contact network is well described, can be a challenging filtering process informed by multiple factors. Techniques are still needed to disentangle these factors, using the different sources of information (evolutionary, immunological, and epidemiological) available to infer likely transmission pathways. Most importantly, we wish to know “how likely is it that individual A infected individual B?”, or “how likely is it that a third unsampled individual was involved in the transmission chain between individuals A and B?”, the key questions in *forensic* or *‘precision’ epidemiology*. The answers to these questions, and transmission pathway reconstruction, are important for gathering information about outbreaks, to shed light on transmission dynamics, and to help infer epidemiological parameters.

Whole genome sequencing (WGS) can be used to detect polymorphisms in a genome with high resolution, and therefore discriminate between closely related strains. Polymorphisms are caused by errors that occur during pathogen replication within the host. Generally these single nucleotide polymorphisms (SNPs) are considered to be neutral in bacterial species within the timescale of disease outbreaks^7^. In the absence of horizontal genetic transfer, tracking these SNPs would be expected to follow the pattern of transmission. In combination with an increasing ability to extract genetic material (either directly from clinical samples or from cultured isolates) and with rapid and minimal processing, large-scale characterization of populations of pathogen genomes is now possible^8–10^. These advances have proven to be transformative for forensic epidemiology, especially when populations can be densely sampled.

The observed genome diversity in a population of pathogens is the result of processes happening at two different scales: the evolution of the pathogen’s genome within the host and its transmission to another host^11^. Both processes are subject to population bottlenecks that could limit strain circulation both within and between hosts^12^. At larger scales, pathogen genotype patterns may be influenced by the contact network; i.e. host social organisation and movement behaviour will determine contact rates within and between host populations. Contact patterns are especially important where disease prevalence and cross-immunity between strains are both high^13^, as this leads to substantial transmission-blocking due to prior infection (and therefore alteration of the effective transmission network by the history of the pathogen itself).

Given the availability of genomic data, the most straightforward approach to describe the relationship between hosts would be based on the genetic clustering of the sampled pathogen strains. However, this would not provide any information about the direction of transmission^11^.

Direction can be estimated by coalescent-based phylodynamic models that infer disease dynamics from an observed genealogy but with only indirect reference to the underlying host demographics^14^. Phylodynamic approaches have been extended further to include different stages of infection and structured host populations^15^, and later an underlying contact network^16^. In the latter case a pairwise coalescent model was embedded within an individual-based stochastic simulation model; this analysis showed that the host contact network can interact with the timing of coalescent events during an epidemic.

Likelihood-based frameworks have been used to identify the plausibility of putative transmission trees. In this case, the likelihood function defines the probability of a transmission tree given temporal (i.e. the date of detection) and pathogen sequence information^17,18^. This approach has been extended further^19^, to show the influence of pathogen within-host dynamics on the relationship between transmission and the phylogenetic tree.

Bayesian inference frameworks have been also used to reconstruct transmission chains, such as in the model developed by Morelli et al.^20^ , which embedded an epidemiological mathematical model in the inference and considered the geographical distance in the transmission probability definition. Another Bayesian method^21^ was used to infer transmission pathways considering the evolutionary and epidemiological processes simultaneously, but did not consider the within-host dynamics of the pathogen, assuming instead that a single dominant strain propagates within and between clusters of hosts. Similarly, the structured-coalescent evolutionary model, SCOTTI^22^ , took a Bayesian approach to reconstruct the transmission events within outbreaks. The SCOTTI framework represents the transmission process as migration events between *populations of pathogens* (i.e. hosts). Li et al.^23^ used a similar method based on particle Markov Chain Monte Carlo (MCMC) to estimate the transmission heterogeneity (i.e. offspring distribution) from incidence time series and pathogen phylogeny.

Maximum parsimony algorithms are also used to estimate transmission events in pathogens outbreaks: they minimise the number of infections consistent with the identified ancestral states in the tree. Romero-Severson et al.^24^ used this criterion with a coalescent HIV model to evaluate the transmission histories of two hosts. They showed that the direction of transmission and the presence of an unsampled intermediary or a common source could be included or excluded depending on the relationship between the two strains and the number of lineages transmitted. The parsimony criteria was also used by Wymant et al.^11^ to develop Phyloscanner, a software tool that can determine transmission pathways from multiple genotypes per infected host. These approaches have produced extremely powerful tools to help disentangle the relationship between pathogen evolution and the infection processes^25^.

However, with the notable exception of SCOTTI (which was applied to *K. pneumoniae* bacterium), these methods have mainly been used to study outbreaks of rapidly evolving RNA viruses such as HIV, Ebola, influenza or foot-and-mouth disease. Campbell et al.^26^ have shown that sequence data for pathogens with lower *transmission divergence* (defined as the number of mutations separating whole genome sequences sampled from transmission host pairs) provide little information about individual transmission events on their own.

*Mycobacterium bovis* is a clonal pathogen, with an extremely low probability of undergoing horizontal genetic transfer at the outbreak scale. Therefore there is a close correspondence between the phylogenetic tree and the transmission network^27^; nevertheless identifying transmission chains is particularly challenging. *M. bovis* is characterized by an extremely slow and highly variable substitution rate, generating low and uncertain levels of genetic diversity, especially when considering small clusters of closely related infections^28–30^. Hence, using these methods, which rely on high divergence, is particularly difficult when attempting to estimate transmission direction for pathogens such as *M. bovis,* the causative agent of bovine/zoonotic/animal tuberculosis (bTB). A further complication arises when dense and/or extensive metadata, which these methods usually require to infer transmission patterns, are not available. This is particularly relevant for pathogens where wildlife are involved (for *M. bovis* this includes badgers, wild boar, or wild ungulates, depending on the specific region^31^), as data on wildlife populations can be both sparse and imprecise.

Here, we developed a method which exploits the Kolmogorov Forward Equations (KFEs) to disentangle the transmission patterns in an infected population. The purpose of the model is to describe the probability of a pair of infected hosts to be in a given state, which was defined by disease progression in both hosts and by the number of single nucleotide polymorphisms (SNPs), assuming that a direct transmission occurred between the pair. This methodology uses the discriminatory power of sequence data to identify transmission pathways and it can be particularly relevant to situations where: (1) SNPs are rare; (2) genetic diversity per transmission generation is low and highly variable; (3) available sequences may be few (here, considering pairs or triplets of sequences); and (4) metadata is limited to the recorded sampling times, as is often the case when data are opportunistically sampled in livestock and wildlife. To maximise the information from such data, we adopted a probabilistic approach to capture both within-host pathogen evolution (i.e. new SNP substitutions) and between-host transmission in a balanced way. This is in contrast to most epidemiological compartmental models, which record the number (or fraction) of individuals in a given infection state (e.g. number of Susceptible, Exposed, and Infectious in the SEI model case), the KFEs describe the probability of the system having a given state, with an exact number of individuals in each infected state^32–34^. We tested this method on a simulated transmission tree and on a bTB infected population of badgers and cattle in Woodchester Park (England). We used this method to test contrasting model assumptions, as well as to assess the likely epidemiological importance of different contact mechanisms.

## Results

### Woodchester Park cattle and badger population

In the present analysis we used the KFEs to calculate the direct transmission probability amongst badgers, cattle, and between the two species. We follow the approach taken by Sharkey^33^, who used the KFEs to describe the infection dynamics at the individual and pairwise level, but here we add states to describe the evolution of the pathogen (i.e. number of SNPs generated after the transmission).

We applied the pairwise KFE model to a previously published dataset describing an endemic *M. bovis* population circulating in cattle (*Bos taurus*) and European badgers (*Meles meles*) in Woodchester Park, Gloucestershire (UK). Since 1977, the population of badgers residing in the Woodchester Park study area has been the subject of an ongoing capture-mark-recapture project^35^. Following an earlier phylogenetic analysis by Crispell et al.^36^, the infected population of cattle and badgers was divided into five clades according to their genetic distance (all isolates within 10 SNPs of one another were considered to be in the same clade). These clades (sub-clades in the case of clade 4) were used as proxies for likely contact network clusters, as we considered clade members to have a higher likelihood of potential infectious contact with one another.

**Figure 1:**
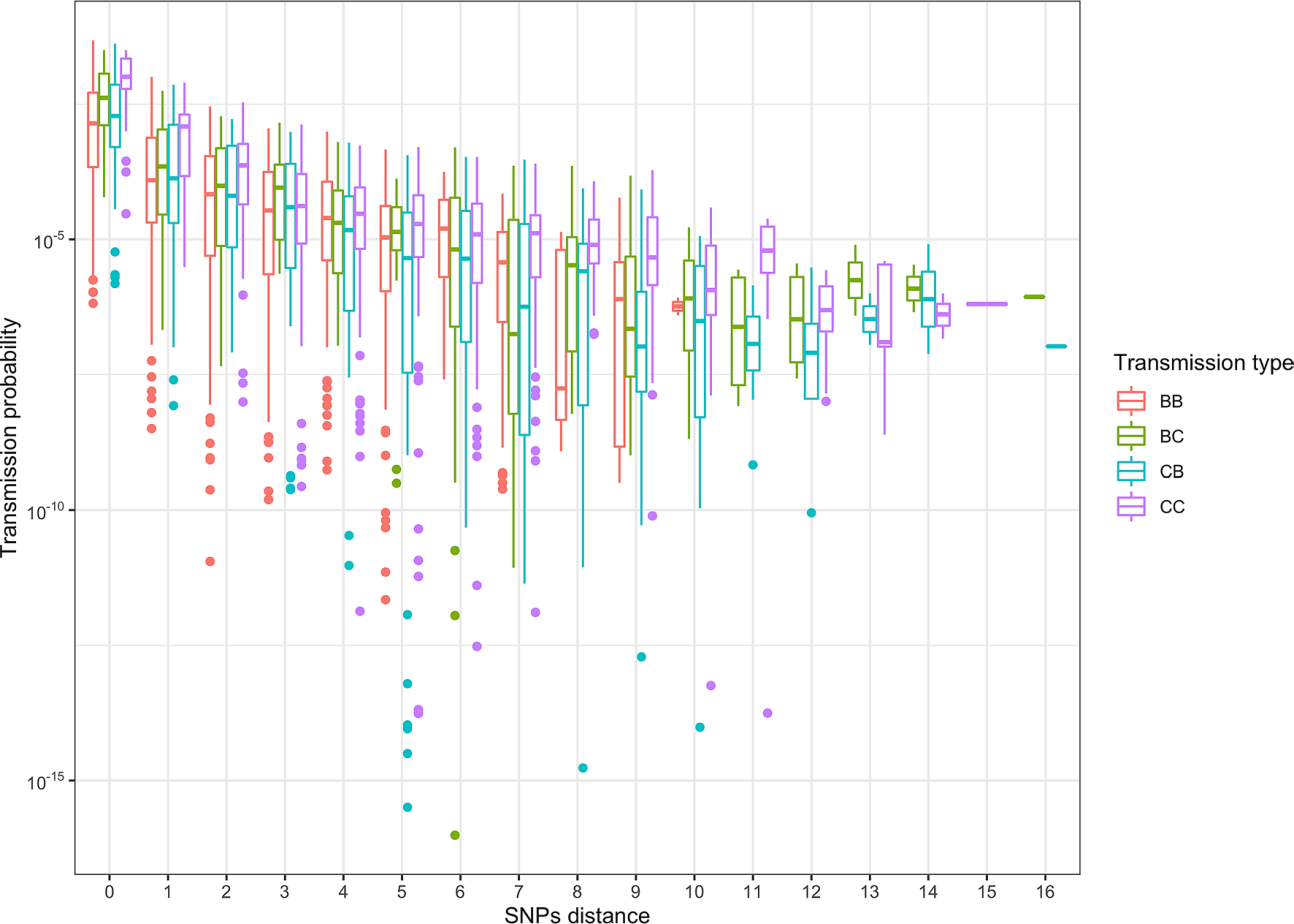
Pairwise transmission probability. The pairwise transmission probability (y-axes) for each host pair versus the SNP distance (x-axis). To calculate the probability we used the following parameters distributions: substitution rate (*μ*), beta-PERT (0.1, 0.31, 0.94 base pair × genome × year); latency period (*1/σ*) beta-PERT (116, 348, 827.5 days); contact rate (*β*) ∈ uniform (0, 0.1) × contact × year.

The transmission probability varied substantially between pairs (median[95% CI] l 0.26 × 10^–4^ [0–0.03]) and was negatively associated with SNP distance (Fig. 1). Although all five clades included strains that have been considered to be closely related (< 20 SNPs distance,^30^), our result showed that even a small SNP difference (up to five) can be useful in discriminating differences in the likelihood of a transmission event between two sampled individuals.

**Figure 2:**
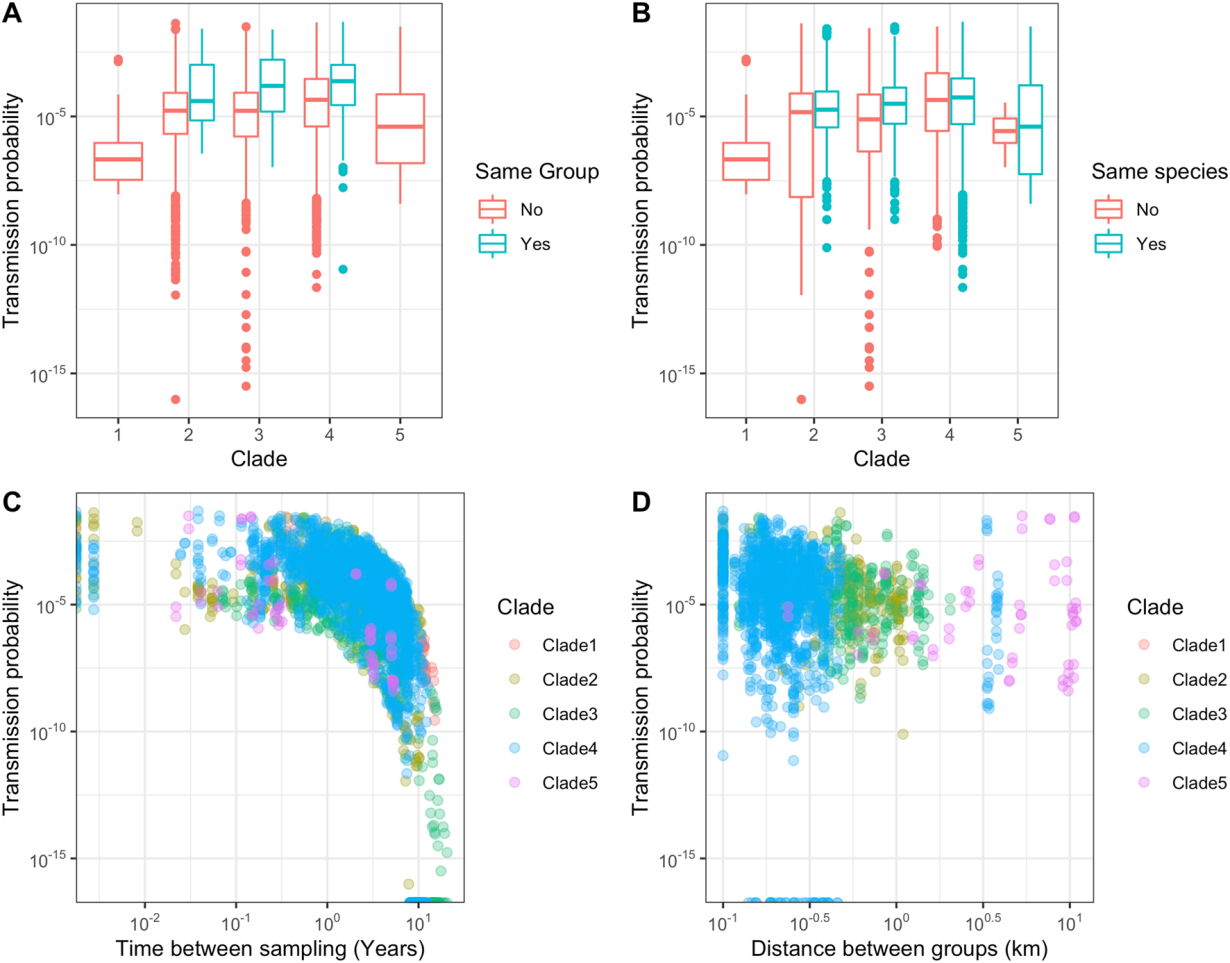
Epidemiological factors influence. Estimated transmission probability (y-axes) versus same group category (**A**), same species (**B**), time between host pair sampling (**C**), and between-groups distance (**D**).

While data on geographic distances between individuals and group (social groups for badgers, herds for cattle) affiliations were available, we deliberately excluded them from the analysis to determine if the effect of distance could be partially recovered by genetic and sample time data alone. As shown in Fig. 2, the proposed method captured differences in the likelihood of transmission between pairs of hosts regardless of whether they belonged to the same social group (i.e. farm or sett, panel A) and species (panel B), although we observed a substantial overlap in the distributions. On the other hand, at the spatial scale considered here (≤ 10 km overall, median 1.48 km) the distance in space did not seem to affect the transmission probability (panel D).

### Transmission trees

**Figure 3:**
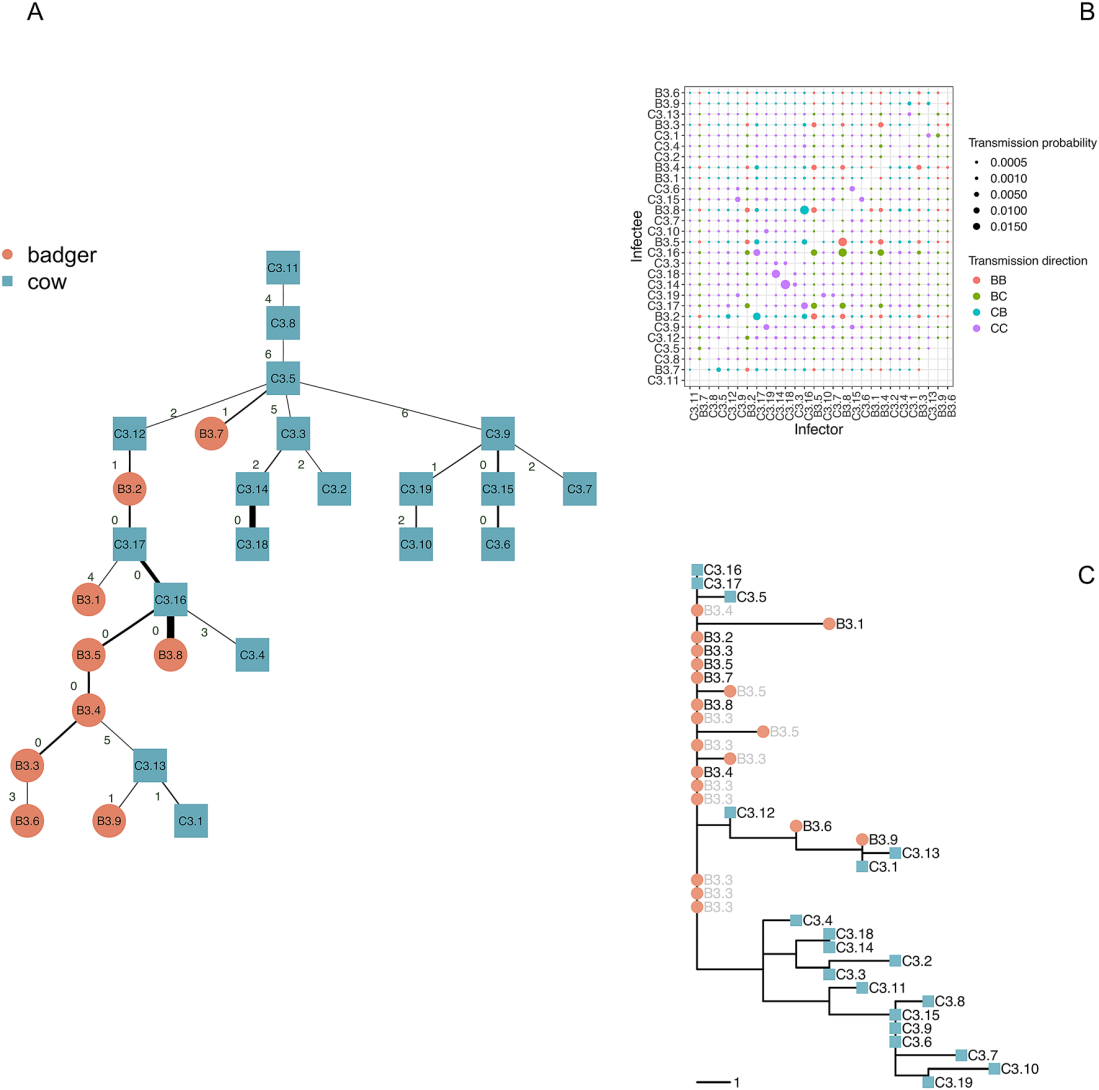
Probability matrix and transmission trees. (**A**) transmission tree for clade 3 (top to bottom). Blue squares represent cattle, while red circles represent badgers. For each represented transmission *i → j*, the edge label indicates the total SNP distance between the isolates sampled in *i* and *j*, while line thickness represents the transmission probability *P*_*(i→j)*_. (**B**) transmission probability for clade 3 pairs, the dot size is proportional to probability, and colour defines the transmission direction (red for badger-to-badger, green for badger-to-cattle, light-blue for cattle-to-badger, and magenta for cattle-to-cattle). (**C**) clade 3 phylogenetic tree (grey-labelled strains were excluded as they were sampled from the same individual, identified by the label number, potentially at different times).

In this section we report the results for clade 3 only (results for clades 1, 2, 4 and 5 can be found in Section 4 in the Supplementary Material). In this case, the median[range] of the stochastic trees likelihood was – 7.85[– 10.14, – 6.91], and Fig. 3A shows the most likely transmission tree for this clade (i.e. corresponding to tree’s likelihood of – 6.91).

Transmission pairs associated with low SNP distance are, as expected, typically characterized by a higher probability (thick lines in Fig. 3A). However the inclusion of sampling time in the inference sometimes indicates that the individual with extra generated SNPs is more likely to be the source than the individual without them (see for example, C3.13 and C3.1, albeit with relatively low probability). As expected, where individuals have little SNP differentiation and similar sampling times, the transmission probabilities are similar and directionality is difficult to discern (e.g. a sub-cluster formed by individuals B3.2, B3.5, B3.8, C3.16, and C3.17; see Fig. 3B). In contrast, where times intervals are long (e.g. individual C3.11 was sampled a decade earlier than others in its clade) the probability of transmitting *M. bovis* to other members of the clade was very low (ranging from 10^–16.1^ to 10^–7.8^), irrespective of SNP distance, indicative of missing infected individuals. As shown in the Supplementary Material (Fig. S3.1), the low and variable substitution rate for *M. bovis* mean that this can be true even for individuals with zero SNP distance but that were sampled more than four years apart, with the probability of an intermediary being higher than for direct transmission, and with the threshold decreased as the number of divergent SNPs increased. Five of the selected transmissions of the tree reported in Fig. 3 (C3.11 → C3.8, C3.8 → C3.5, C3.5 → C3.9, B3.4 → C3.13, and C3.5 → C3.3) had a difference in sampling times (Δt) that was higher than the threshold shown in the Supplementary material (Fig. S3.1), thus indicating transmission chains more likely to have been mediated by missing hosts than via a direct route.

### Alternative bottleneck model

In the model described above we assumed that SNP substitutions within an infected host can occur during latency (i.e. after infection but before infectiousness onset), so that the pathogen population bottleneck occurs at the point of infection, where only a very small number of bacteria are transmitted. It is however possible that the bacteria only replicate appreciably once active infection has started, and that in the latent stage, pathogen evolution and therefore substitution rates are low or zero. Although this issue has not been explored yet for *M. bovis,* controlled experiments with the closely related *M. tuberculosis* showed no variation in substitution rates in the latent and active disease stages in macaques^37^; a further study in a population of infected humans found low growth and substitution rates during latency^38^. While the former observation would imply a pathogen population bottleneck at the infection stage (i.e. when the host state changes to Exposed ), the latter implies a bottleneck at the end of the latency stage (i.e. Infectious state) and thus no definitive model for the within-host evolution bottleneck exists.

Here, we explored the implications of different population bottleneck models by calculating the pairwise transmission likelihood with two alternative within-host models. Specifically, we allowed divergent SNPs to appear either from the point of infection, or at the infectious (I) stage only (i.e. setting the mutation rate for Exposed individuals to 0) for one of the clades (clade 3).

**Figure 4:**
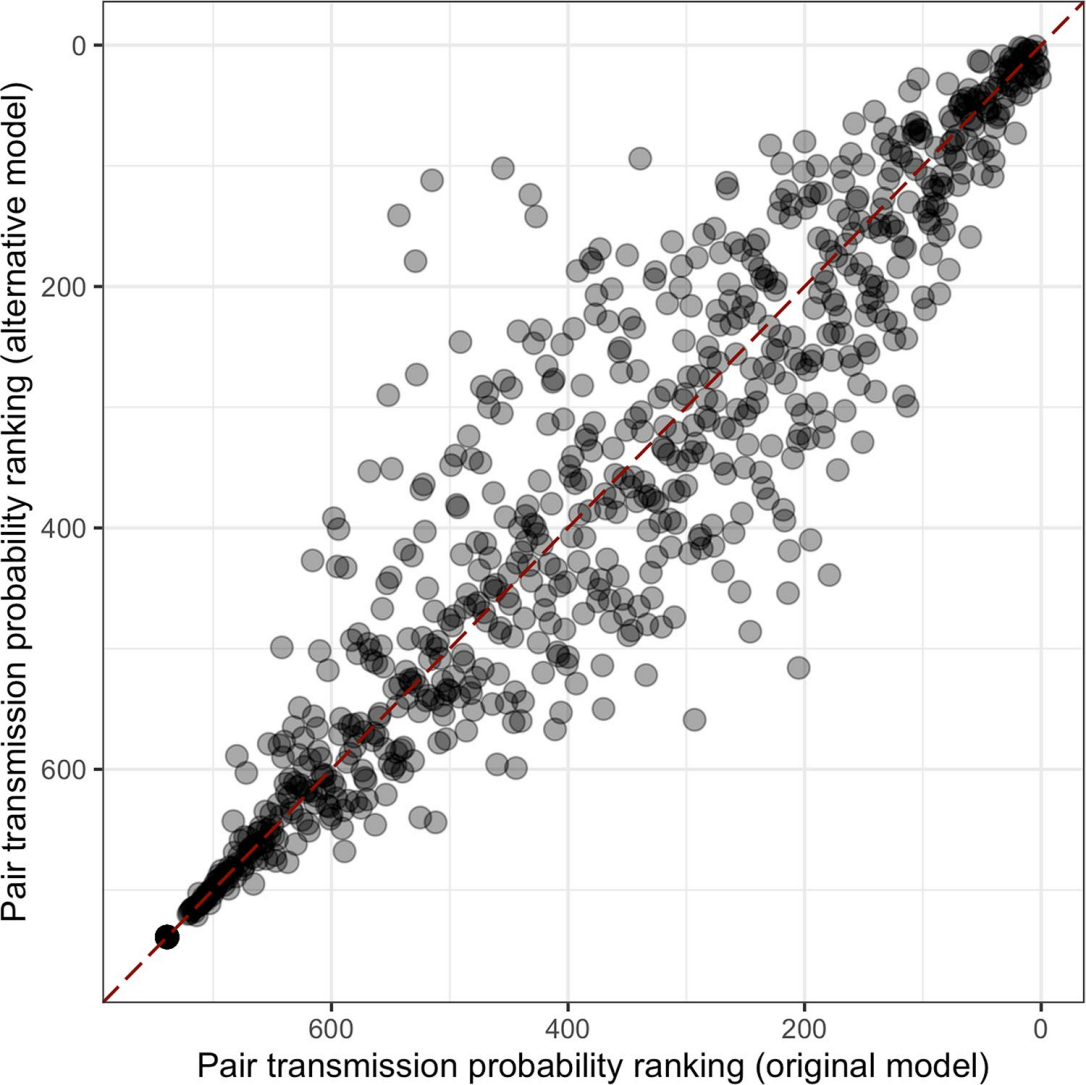
Transmission probabilities ranking with different bottleneck assumption. Comparison of the pairwise transmission probabilities ranking for each hosts pair using the original model (SNPs substitution allowed during the exposed stage, pathogen population bottleneck at infection) and an alternative within-host model (SNPs substitution allowed at infectious stage: pathogen population bottleneck after latent stage).

**Figure 5:**
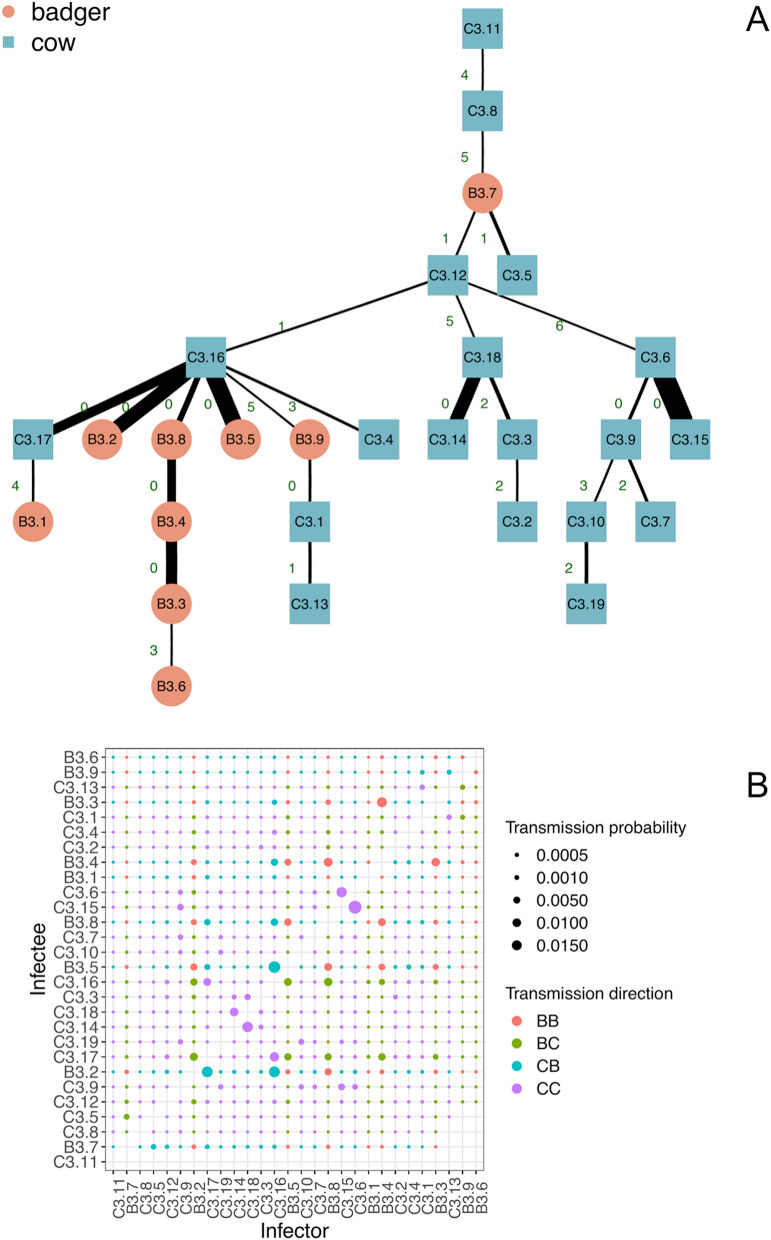
Transmission tree with alternative pathogen bottleneck model. Transmission tree (**A**, top to bottom) and transmission probability matrix (**B**) for clade 3, assuming an alternative model where SNPs substitution is allowed during the infectious period only (see Fig. 3 caption for full description).

We showed that although the average transmission likelihood did not changed, and the lower estimates did not change much using the alternative model, for many of the more likely transmission pairs there was a substantially stronger effect (Fig. 4). The resulting tree and matrix are respectively reported in Fig. 5A,B. They show that two selected but unlikely transmission pairs were consistent in both models (e.g. C3.11 → C3.8 and C3.17 → B3.1). Differences in the likelihood of transmission events were also identified even when the strains were closely related (e.g. C3.15 was infected by C3.9 with the first model, and by C3.6 with the alternative one). These differences were due to the ability of this approach to discriminate between host pairs with similar SNPs distance (0 or 1). This was particularly evident for the triad formed by C3.6, C3.9 and C3.15, all at 0 SNPs distance between one another. While the original model inferred the transmission chain C3.9 → C3.15 → C3.6, the alternative model inferred that C3.6 infected both the C3.9 and C3.15. The triad formed by B3.9, C3.1 and C3.13 could be considered similarly, since the original model inferred C3.13 → C3.1 and C3.13 → B3.9, whilst the alternative one inferred B3.9 → C3.1 → C3.13. However, some clusters where SNP distances were close were robust to the bottleneck assumption, in particular those formed by C3.2, C3.3, C3.18, and C3.14, and by C3.6, C3.7, C3.9, C3.10, C3.15, and C.19, although in the latter the position of many hosts and the inferred direction of transmission differed. These clusters were also consistent with the phylogenetic tree shown in Fig. 2, bottom-right panel.

## Discussion

In infectious disease epidemiology, the importance of considering processes at different scales for disentangling the dynamics of outbreaks is well recognised^39,40^. Recent improvements in techniques to obtain pathogen genomic information have resulted in a number of advanced methods that can use genomic and epidemiological data to disentangle the roles of different contributing processes, including the infectious contact network^15,25,41–43^. However, while many of these methods are suited to the analysis of rapidly evolving pathogens such as RNA viruses (i.e. HIV, SARs, Foot-and-Mouth Disease, Ebola), applying the same methods to slowly evolving and spreading pathogens is more challenging, due to the low signal-to-noise ratio (i.e. in SNPs) when comparing different strains^26,39,44^. Chronic bacterial diseases are usually characterised by long and variable latency periods that increase uncertainty around the timing of transmission events, and also contribute to partially sampled outbreaks, as the infected host can be removed from the population by other causes before exhibiting the symptoms of infection^45^.

Here, we have presented a model to study infectious diseases dynamics at a forensic level, combining within-host evolution of *M. bovis* and between-host transmission, which relies on genomic information and basic epidemiological data only. Since pathogen evolution and infection processes are intimately related, we showed that combining the sampling time (often the only epidemiological information available), whole genome sequence data for the bacteria, and a simple model for disease progression (here incorporating an exposed stage before infectiousness) can help discriminate amongst possible transmission pathways. This was possible even amongst groups of individuals infected with highly similar strains (i.e. fewer than five SNPs between them), even though it was not always possible to definitively identify who-infected-whom. As expected, a change in the within-host scale dynamics and therefore the pathogen population bottleneck can influence this result. Thus where information about within-host pathogen dynamics is available, these data should be used to inform the model. Where such data are not available, our method can be used to identify when inferred transmission pathways are robust to different bottleneck assumptions.

Our method exploits a crucial feature of the combined genetic and temporal data: while the time of sampling is useful to identify the probability boundaries for a possible transmission pair (broadly speaking, the farther apart in time for the sample dates, the less likely a transmission event), the genetic distance between the bacterial genomes embeds the combination of temporal distance and transmission distance over the network of potential contacts, parsed by the rate of pathogen within-host evolution. The inclusion of a latent stage further informs the inference (for example, the inclusion of the latent stage reduces the probability of infectious contact if the sample dates, and therefore the infection dates, are too close to each other), and therefore an informed model should be better able to estimate inter-generation times and times of infection than approaches that do not consider the epidemiology.

Unlike many other approaches that consider the population as a whole, here we adopted a pairwise approach. A similar forensic approach was used by Campbell and colleagues in order to develop *outbreaker2*, which considered temporal, contact network, and genomic data to infer transmission events, although it did not consider the transmission process as we do here^46^. While on the one hand pairwise approaches substantially simplify the computation, on the other hand they introduce dependency problems that might bias the transmission likelihood calculations^46^. Our method is reliant on unbiased sampling, and it does not consider any higher order interactions that might be embedded in the system. Such interactions might be important if, for example, there are substantial interactions between the infection pressures from two individuals on a third. Counterbalancing this is the exactness of the KFE approach.

While earlier results show that in the Woodchester Park badger dataset geographical distance between *M. bovis* isolates is an important predictor of their genetic distance^36^, our method did not capture the effect of distance on the pairwise transmission probability. This might be a consequence of the studied spatial scale: only for clade five (which included mostly cattle) between-group distances were above 5 km, while the median distance overall was 1.48 km only. While this sits slightly above the badger territory size observed in the area^47^, individuals might roam longer distances^48^. Since we considered pairwise interactions only, it may be that the relationship with distance would be recovered in an analysis of longer transmission chains. As expected, the social group provided a strong signal. These results suggest that, for this context, contact networks could be better informed by social interactions than by spatial distance. These results were obtained despite our methods suggesting many missing links in the obtained transmission trees, as we were able to detect the branch in the transmission trees where it is more likely that one or more infected hosts were not sampled. It is important to consider that the number of unsampled individuals is not known, and so it is always possible that an unsampled individual was directly involved. However, the density of sampling of the Woodchester Park badger population was high, with animals being trapped and tested for *M. bovis* infection on average twice every year^36^. Even considering the relatively low sensitivity of the testing approach^49^, we would still expect that our highly likely pairs would have more closely related infections compared to our less likely pairs. The fact that we were able to identify circumstances when an intermediate host was likely is particularly notable given the low level of diversity in *M. bovis* genomic data. Furthermore, we only used up to five SNPs in our estimates.

The analyses presented here follow a simplistic representation of transmission dynamics, but could easily be expanded within the KFE framework, albeit at increased computational cost. For example, we did not account for events other than the sequence sampling. Since UK cattle are regularly tested for bTB as part of the routine national control program, we could include the probability of being infected but not detected on the date of a negative test prior to a positive one. Other epidemiological data (distance, population groups, species, etc.) could also be explicitly embedded, and might be used to identify, for example, differences in estimated parameters for different species combinations.

In our calculations, we considered values of SNP difference up to five. The choice of this low SNP threshold was mainly for computational reasons, as for every extra SNP the size of the matrix used to solve the KFEs increases geometrically, thus slowing the calculations. However, Walker and co-authors^50^ showed that for human TB five SNPs is likely sufficient to discriminate between likely and unlikely transmission events. Our results showed a similar pattern with the probability dropping below 10^–5^ past the fourth SNP (Fig. 1). Furthermore, this result also highlights how direct transmission between hosts in different clades is even more unlikely, since their SNP distance is higher than 10. One potential limitation of this study is the absence of a well-defined contact network between sampled infected hosts, which would have further reduced the number of potential transmission pathways. In the absence of these data, given the slow substitution rate of *M. bovis,* dividing groups based on their bacterial genetic distances proved to be a useful first step in conducting this analysis.

In order to build the transmission tree we used a simple but intuitive algorithm compared to other methods in the literature (^25^, and references therein), which allowed us to identify the poorly supported transmissions where unsampled infected hosts were more likely to be involved. A more sophisticated algorithm for building the transmission trees would provide additional insight but at computational costs.

The objective of this study was to disentangle the interactions between processes happening at the within-host scale and at the population level, in order to refine the representation of the potential transmission network, compared to considering SNP distance alone when limited metadata and only a few samples are available. By using the KFEs in a novel analytical approach, we have shown how accounting for the evolution of *M. bovis* strains and incorporating an epidemiological model can successfully distinguish between many likely and unlikely transmission scenarios, despite the low genetic variability observed due to the slow and variable substitution rate that characterizes *M. bovis*, and with only a limited number of samples. Our KFE method is flexible and precise, and could be applied to other chronic infections where identifying who infected whom can be difficult, such as Johne’s disease (*Mycobacterium avium paratuberculosis* infection) in cattle, or leprosy in humans, contributing to an improved understanding of the role of within-host evolution and epidemiological dynamics in inferring contact patterns.

## Methods

### The pairwise Kolmogorov Forward Equations (KFEs) with within-host dynamics

The purpose of the following model is to describe the probability of pair infected hosts to be in a given state of infection progression, assuming that a directional transmission event occurred between the two. We specify a three-state Susceptible-Exposed-Infectious (SEI) model as being appropriate to the epidemiology of *M. bovis* infection dynamics^45,51–56^. Here, *Susceptible* individuals become *Exposed* (but not infectious) after a successful transmission from an *Infectious* individual, and then move to an *Infectious* state after the latency period. The transmission rate and the E to I progression rate are represented by β and σ, respectively (thus 1/σ is the average duration of the E stage).

We consider within-host evolution in parallel with disease progression in the dynamic model, in order to account for differences between the observed and transmitted lineages of bacteria in a host^10^ and to explore the potential impact of variation in population dynamics at the within-host level. For simplicity we consider all nucleotide transitions to be equally likely (i.e. rate of A → T is the same as rate of C → G, etc.).

Strain evolution is modelled as a dynamic process occurring simultaneously alongside the infection progression, with the number of SNPs indicated by the superscript *k* (thus the full host state is denoted as ${n}_{i}^{k}$, with *n*_*i*_ representing the epidemiological status) and these SNPs generated at a fixed *substitution rate* (μ). Here, we refer to the strain harboured by a host before it is transmitted to another as *ancestral*, and it is denoted by *k* = *0* (i.e. ${n}_{i}^{0}$). When *k* > *0* it denotes the number of SNPs (${n}_{i}^{1}$, ${n}_{i}^{2}$, ${n}_{i}^{3}$ …) in the mutant strains. Following previous researchers, we have labelled the extra SNPs in a given sampled mutant strain as *divergent SNPs*^26^. These SNPs does not include the substitutions occurring before the transmission, as we consider these common to both hosts strains.

In the case of a pair of hosts, the KFE system is defined as follows:

1 $$\left\{\begin{array}{l}\frac{{dP}_{{E}^{0}S}}{dt}= -\sigma {P}_{ES}\\ \frac{{dP}_{{I}^{0}S}}{dt} = \sigma {P}_{ES}-\beta {P}_{{I}^{0}S}\\ \frac{{dP}_{{I}^{0}{E}^{0}}}{dt}= {\beta {P}_{{I}^{0}S}-(\sigma +2\mu )P}_{{I}^{0}{E}^{0}}\\ \frac{{dP}_{{I}^{0}{I}^{0}}}{dt} = {\sigma {P}_{{I}^{0}{E}^{0}}-2\mu P}_{{I}^{0}{I}^{0}}\\ \frac{{dP}_{{I}^{1}{I}^{0}}}{dt} = {\mu {P}_{{I}^{0}{I}^{0}}-2\mu P}_{{I}^{1}{I}^{0}}\\ \frac{{dP}_{{I}^{0}{I}^{1}}}{dt} = {\mu {P}_{{I}^{0}{I}^{0}}-2\mu P}_{{I}^{0}{I}^{1}}\\ \frac{{dP}_{{I}^{1}{I}^{1}}}{dt} = {\mu {(P}_{{I}^{0}{I}^{1}}+{ P}_{{I}^{1}{I}^{0}})-2\mu P}_{{I}^{1}{I}^{1}}\\ \dots \end{array}\right.$$

Here, ${P}_{{{n}_{i}^{k}n}_{j}^{l}}$ is the probability of *A* being in status *n*_*i*_ and infectious and *B* being in status *n*_*j*_, with *k* and *l* divergent SNPs, respectively. For a full derivation of the KFEs see the Supplementary material S1.

Using this system, we calculate the exact probability for two hosts in any possible combination of infection states and for any number of divergent SNPs. Hence, assuming that host *A* was first exposed to the infection at time *t*_0_, host *B* was infected by the former, and pathogen strains from both hosts were sampled at time *t*_*T*_ (= *t*_*A.*_= *t*_*B*_),we can numerically solve the system (1) from *t*_*0*_ and *t*_*T*_ to obtain the transmission probability:

2 $${P}_{A\to B}= {P}_{{I}^{k}{E}^{l}}\left(t\right)+ {P}_{{I}^{k}{I}^{l}}\left(t\right)$$

with *t* = *t*_*T*_ – *t*_*0*_.

Where the sampling time differs (*t*_*A.*_≠ *t*_*B*_), we calculate the transmission probability in two steps. Assuming *t*_*A*_ < *t*_*B*_, we solve the system (1) from time *t*_*0*_ until A was removed at *t*_*A*_ (removal/sampling time). This result is then inserted into a single-host KFE model which is solved between *t*_*A*_ and *t*_*B*_ (for the one-host KFE system see Supplementary material S1). An analogous reasoning holds for the case *t*_*B*_ < *t*_*A*_.

The equations in (1) are linear, and so in principle it is trivial to find the solution of the ${P}_{A\to B}$. However, given the complexity of the system and number of equations increasing with the number of SNPs considered, we opted for a numerical solution implemented in R^57^, with package *deSolve*^58^.

### Application of the pairwise KFE model

The badger population was sampled in the context of a long-term capture/recapture project in Woodchester Park^35^, while their *M. bovis* strains were sequenced for a previous study^36^. Clinical samples were taken from each captured badger and undergo microbiological culture to attempt to isolate *M. bovis*. Whole genome sequences from 191 isolates collected since 2000 were made available for the present study. In addition, 69 M*. bovis* sequences sourced from cattle on neighbouring (within 10 km) farms since 1988 were also made available. These farms were sampled as part of the routine bTB control and eradication program, and the associated testing information is stored in the APHA cattle testing (SAM) database^59^.

As the contact structure between infected hosts was not known, we subdivided our analysis based solely on the available genetic information. Following Crispell et al.^36^ we divided the Woodchester Park infected population into five main clades (see Table 1). The clades were originally defined as containing highly related *M. bovis* sequences (all isolates within 10 SNPs of one another) sourced from cattle and badgers and we used this information as a proxy for evidence of a high likelihood of contact. We conducted our analysis on each clade separately, except for the very large clade 4 which was further divided into sub-clades for computational reasons. We divided the clade into further seven sub-clades of size ranging between 8 and 31 highly related sequences, or 6 to 22 hosts, in order to reduce the number of investigated pairs from 1700 to 11,3422 (see Table S4.2 for detailed SNPs distances). For badgers yielding more than one *M. bovis* sequence, we only considered the most recent one (the unused sequences are labelled in grey in panel C of Fig. 3 for clade 3, and in Supplementary Material Section 4 figures for other clades). We then counted the differences between each pair of strains, to obtain the number of divergent SNPs. This process was applied to all pairs of individuals present in each clade (or sub-clade). For the sake of simplicity, we labelled all individuals according to their species (B for badgers and C for cattle), main clade (1 to 5), and a sequential integer number. For each pair of sampled individuals in each clade (or sub-clade), we independently calculated the probability that one host infected the other, considering both transmission directions. After defining the epidemiological model (e.g. SEI), the only data needed to perform the probability calculation were the timings of sampling for the two hosts (*t*_*A*_ and *t*_*B*_), and the observed divergent SNPs (*k* and *l*) at the respective time of sampling.

**Table 1:** Woodchester Park (UK) *M. bovis* sequences and clades (based on Crispell et al.^36^).

Clade	Sub-clade	# *M. bovis* sequences	# *M. bovis* sequences from badgers	# *M. bovis* sequences from cattle	# hosts	# badgers	# cattle	Sampling year range
Clade 1	–	11	4	7	8	1	7	1988–2003
Clade 2	–	32	10	22	27	5	22	2002–2013
Clade 3	–	39	20	19	28	9	19	1993–2013
Clade 4	(total)	170	156	14	107	93	14	2000–2012
Clade 4	a	27	24	3	16	13	3	2000–2011
Clade 4	b	29	27	2	18	16	2	2002–2011
Clade 4	c	24	23	1	15	14	1	2000–2011
Clade 4	d	28	26	2	22	20	2	2000–2012
Clade 4	e	10	8	2	6	4	2	2003–2010
Clade 4	f	19	17	2	11	9	2	2005–2011
Clade 4	g	33	31	2	19	17	2	2000–2008
Clade 5	–	8	1	7	8	1	7	2008–2013

As the epidemiological parameters for bTB are highly uncertain, instead of using single values we drew them from distributions informed by the recent literature. We then allowed variability in the probability calculation by moving each parameter according to a Gaussian kernel (mean equal to the previous iteration values, sd = 5%) for 1000 iterations, each time calculating the pairwise transmission probability. We finally selected the combination of parameters which resulted in the highest transmission probability. Given that potential differences in infection progression in badgers vs. cattle are unknown, we considered the same set of parameters for both species. A beta-PERT distribution was used for the substitution rate (*μ*), with mode, minimum and maximum respectively set to 0.31, 0.1 and 0.94 base pair × genome × year. These values corresponded to the average, minimum, and maximum of literature estimates from other systems^28,60–62^. We assigned a similar distribution to the latency period (*1/σ*) based on the published literature for data relevant to the geographical area (Southern England). In this case we used a beta-PERT distribution, with mode, minimum and maximum respectively set to 348, 116 and 827.5 days^54,55^. Finally, we used a uniform distribution for the contact rate: *β* ∈ *U*(0, 0.1) × contact × year. This was set to include the transmission rate estimates in^54,55^, but widened to account for the fact that the pairwise infection rate in our model assumes that contact exists (i.e. the population level parameters consider both probability of contact and infection rate given that contact). We defined a constant death rate, based on the observation that less than 0.1% of individuals would survive past 8-years of age (*maximum lifespan*) for both cattle^63^ and badgers^48^.

### Likely transmission trees

In order to identify the most likely transmission trees, we assigned a weight (*W*_*AB*_) to each potential within-clade transmission between *A* and *B*, calculated by dividing the transmission probability P_A→B_ by the sum of all transmission probabilities in the same clade. Then, we built the tree by sequentially drawing transmission pairs with probability depending on their weights, until the transmission tree was completed (i.e. only one host was left without a transmission source, representing the tree’s root). At each stage, and assuming only a single source for each infection, we discarded all implausible transmissions resulting from previous selections (i.e. if a selected transmission was from *A* to *B*, then *B* to *A* was ruled out, as well as the transmission from other hosts to B), and also avoiding loops. For each clade we built 10,000 stochastic transmission trees, and for all trees, we calculated the *tree likelihood* (*L*) as follow:

3 $$L=\frac{\sum_{A}{\sum }_{B\ne A}{K}_{AB}}{N},$$

where *N* is the total number of transmissions in the tree, and

4 $${K}_{AB}=\left\{\begin{array}{ll}log({P}_{AB})& if \,transmission \,A\to B \,was \,selected\\ 0& otherwise\end{array}.\right.$$

## Supplementary Information

Supplementary Information.

## Data Availability

All data used in the present study were previously generated and/or collected, therefore this study did not use animals. All WGS data used for these analyses have been uploaded to the National Centre for Biotechnology Information Short Read Archive (NCBI-SRA: PRJNA523164). Because of the sensitivity of the associated metadata, only the sampling date and species will be provided with these sequences.
